# Six-Year Biochar Experiment Reduces Soil N_2_O Emissions in *Eucalyptus* Plantations: Associations with Microbial N-Cycle Genes 

**DOI:** 10.3390/microorganisms14071519

**Published:** 2026-07-12

**Authors:** Yunhuang Luo, Yuyi Shen, Hao Shi, Qiumei Teng, Guangping Xu, Liangliang Huang, Junzhi Chu, Jialin Liao, Denan Zhang, Kechao Huang, Yingjie Sun, Zhiwen Tan, Yu Cao

**Affiliations:** 1 College of Environmental Science and Engineering, Guilin University of Technology, Guilin 541000, China; 2120240612@glut.edu.cn (Y.L.); llhuang@glut.edu.cn (L.H.);; 2 Guangxi Key Laboratory of Plant Conservation and Restoration Ecology in Karst Terrain, Guangxi Institute of Botany, Guangxi Zhuang Autonomous Region and Chinese Academy of Sciences, Guilin 541000, China; shihao@gxib.cn (H.S.); tqm1907@163.com (Q.T.); denanzhang@126.com (D.Z.); kchuanggxib@163.com (K.H.); syj@gxib.cn (Y.S.); 3 Guangxi Key Laboratory of Functional Phytochemicals and Sustainable Utilization, Guangxi Institute of Botany, Guangxi Zhuang Autonomous Region and Chinese Academy of Sciences, Guilin 541000, China; 4 Guangxi Guilin Urban Ecosystem Observation and Research Station, Guilin 541000, China; 5 College of Life Sciences, Guangxi Normal University, Guilin 541000, China

**Keywords:** biochar, nitrous oxide (N_2_O), functional gene, nitrification, denitrification, *Eucalyptus* plantation

## Abstract

Nitrous oxide (N_2_O) is a major greenhouse gas, and terrestrial ecosystems are among the primary sources of its emissions. Biochar is recognized as an effective soil amendment for mitigating N_2_O emissions, but its long-term residual effects and microbial mechanisms in subtropical plantations remain unclear. Therefore, this study evaluated the residual effects of *Eucalyptus*-derived biochar on soil N_2_O emissions six years after a single application and explored associations with nitrogen cycle functional genes. A field experiment was conducted in a *Eucalyptus* plantation in northern Guangxi with biochar applied at six rates (0–6% *w*/*w*). Soil N_2_O fluxes were measured in the fifth and sixth years (2022–2023); soil chemical parameters, soil enzyme activities, and N_2_O-related microbial functional genes (*amoA*, *nirK*, *nirS* and *nosZ*) abundance were analyzed. Biochar application significantly reduced ammonium nitrogen content but enhanced nitrate nitrogen content. Urease, protease, and sucrase activities increased, while nitrate reductase, nitrite reductase, and hydroxylamine reductase activities decreased. Furthermore, quantitative analysis revealed substantial variations in functional gene abundances. The abundance of ammonia-oxidizing archaea (AOA-*amoA*) exhibited a unimodal response, whereas ammonia-oxidizing bacteria (AOB-*amoA*) showed a robust dose-dependent accumulation. Notably, annual N_2_O emissions were suppressed by up to 35.2%, driven by a 3.4-fold increase in *nosZ* gene abundance and a significant reduction in the (*nirK* + *nirS*)/*nosZ* ratio. This mitigation was attributed to enhanced N_2_O consumption by *nosZ*-harboring denitrifiers and reduced heterotrophic ammonia oxidation. Overall, these findings highlight the pivotal role of long-term organic amendments in steering nitrogen transformation pathways, providing a theoretical basis for sustainable soil management in subtropical plantations.

## 1. Introduction

Greenhouse gases, such as carbon dioxide (CO_2_), methane (CH_4_), and nitrous oxide (N_2_O), increase Earth’s surface temperature by enhancing the greenhouse effect [1]. N_2_O is one of the major greenhouse gases with a retention period of approximately 114 years in the atmosphere, indicating persistence in the environment for a long time [2]. Although N_2_O accounts for only about 0.03% of total greenhouse gas emissions, its global warming potential is approximately 265 times that of CO_2_ [3]. Since industrialization, the concentration of N_2_O in the atmosphere has increased by more than 20%, leading to ozone depletion, ecosystem imbalances, and intensified greenhouse effects. These changes and its impact on global climate change have attracted much attention [4]. Atmospheric N_2_O concentrations, which are primarily driven by anthropogenic activities, are rising continuously [5]. According to the recent high-resolution projections building on the global N_2_O budget, these anthropogenic emissions are rising at an accelerating pace, necessitating more robust mitigation frameworks including stricter N policies [6].

More than two-thirds of terrestrial N_2_O emissions are driven by microbial nitrification and denitrification pathways mediated by soil bacteria and fungi, which are primarily driven by the excessive use of nitrogen fertilizers [7]. The nitrification process in soil is primarily regulated by ammonia-oxidizing archaea (AOA) and ammonia-oxidizing bacteria (AOB), which typically exhibit distinct niche differentiation and adaptive thresholds under varying substrate availability [8].

In addition, the functional genes *nirK*, *nirS*, and *nosZ* play crucial roles in the denitrification process [9], with the (*nirK* + *nirS*)/*nosZ* ratio serving as a key proxy for net N_2_O reduction efficiency. Therefore, exploring the interrelationships among soil physical and chemical properties; enzyme activities; AOA and AOB abundance or functions; and the genes *nirK*, *nirS*, and *nosZ* is essential. Research on the biological mechanisms underlying soil N_2_O emissions is crucial for addressing global climate change. Biochar, recognized for its cost-effectiveness, stability, and environmental remediation potential, has emerged as a promising soil amendment material. In recent years, it has been extensively used for soil remediation purposes [10]. Studies have demonstrated that biochar can significantly reduce N_2_O emissions in some agricultural and forest soils [11]. However, other studies have reported highly variable effects, ranging from no significant net reduction [12,13] to even increased emissions under specific conditions [14]. Most of these studies were conducted over short periods (typically <1 year) or under indoor cultivation conditions. Given that short-term experiments often fail to account for critical long-term processes such as biochar aging effects and the accumulation of soil organic matter, there is a lack of validation through long-term field trials of biochar application, particularly regarding how biochar continuously regulates microbial functional groups over extended temporal scales. The influence of biochar on soil N_2_O emissions depends on various factors, such as biochar type, experimental duration, soil characteristics, and vegetation type. Therefore, the long-term effects of biochar application on N_2_O emissions still require further investigation [15].

*Eucalyptus* is widely distributed in the subtropical regions of China and is one of the important afforestation tree species in China [16]. It plays an indispensable role in ecological restoration, increases forest coverage, and enhances wood processing [17]. However, short rotation cycles and intensive management practices have caused ecological problems, such as reductions in soil fertility, soil acidification, and alterations in soil microbial community structure. These changes, especially the imbalances within nitrogen cycling microbial networks, may contribute to increased N_2_O emissions from *Eucalyptus* plantations [18,19,20,21].

However, knowledge regarding the residual effects of a single biochar application on soil N_2_O emissions over extended temporal scales in subtropical *Eucalyptus* plantations remains scarce. To address this gap, this study leveraged a six-year in situ field experiment conducted in a *Eucalyptus* plantation in northern Guangxi to investigate the residual effects of a single *Eucalyptus*-derived biochar application on soil N_2_O emissions. It is hypothesized that biochar reduces soil N_2_O emissions through dual mechanisms involving soil chemical improvement and microbial reconfiguration. The specific objectives were as follows: (1) to evaluate the long-term effects of biochar on soil properties; (2) to quantify the residual mitigation of soil N_2_O emissions six years after a single biochar application; and (3) to elucidate the microbial mechanisms underlying these changes.

## 2. Materials and Methods

### 2.1. Sampling Area and Experimental Setup

The study area was situated in the state-owned Huangmian Forest Farm (24°37′25″–24°52′11″ N, 109°43′46″–109°58′18″ E) at the junction of Luzhai County (Liuzhou City) and Yongfu County (Guilin City) in Guangxi. This region is characterized by a subtropical climate with an annual average temperature of 19 °C and rainfall of 1750–2000 mm, primarily concentrated between April and August. The forest farm is characterized by low mountains and hills and predominantly contains red soil and mountain yellow–red soil. The physicochemical properties of the soils are shown in Table S1 in the Supplementary Materials.

This study was an extension of an in situ biochar application experiment established in March 2017 in a *Eucalyptus* plantation [22,23]. Biochar was produced from *Eucalyptus* branches collected from the forest farm and surrounding areas via pyrolysis at 500 °C for 2 h under anaerobic conditions (Jining Dehanqi Mechanical Engineering Technology Co., Ltd., Jining, China). The basic properties of the biochar are presented in Table S2 in the Supplementary Materials. The prepared biochar was then milled and screened to a 2 mm sieve to ensure homogeneous particle dimensions. The experimental plots were selected from a typical *Eucalyptus* plantation with similar parent materials, altitudes, and relatively gentle topographies. Biochar was applied following the protocols outlined by [24,25], with application rates controlled based on the mass percentage of biochar to dry soil weight. Biochar application ratios comprised six treatments: CK (0%), T1 (0.5%), T2 (1.0%), T3 (2%), T4 (4%), and T5 (6%). Each treatment contained three replicates, making a total of 18 plots of 8 m × 8 m each, with 1 m buffer zones between adjacent plots. The 18 plots were arranged in a randomized complete block design with three blocks. Blocks were established to account for topographic variation across the site, and each treatment was randomly assigned to one plot within each block. Prior to application, all the plots were ploughed to a depth of 30 cm, and biochar was thoroughly mixed into the tilled soil at the designated ratios in a single application. The control plots were tilled similarly without the addition of biochar. No additional fertilizer was applied during the experiment, and biochar was applied only once to observe its long-term effects.

### 2.2. Sample Collection

Soil samples were collected from the *Eucalyptus* plantation in northern Guangxi in March 2023. Five representative sampling points per plot were selected following an S-shaped sampling design. Soil samples were collected from the surface layer (0–10 cm) using a stainless steel corer. Immediately after collection, the samples were placed in sterile polyethylene bags, stored in a sealed ice container, and transported to the laboratory without delay. Upon arrival at the laboratory, samples were stored at 4 °C and −20 °C in a dedicated refrigerator until further analysis. Each sample was subsequently divided into two subsamples: fresh sample for microbial community analysis and air-dried sample for physicochemical characterization. Soil surface temperature, soil water content, and pH were continuously monitored from January 2022 to December 2023, corresponding to the fifth and sixth years after a single biochar application.

### 2.3. Soil Physicochemical Property Analysis

Soil surface temperature (ST) and soil water content (SWC) were measured in situ using a portable three-parameter probe (TDR-350, Jinkai Co., Ltd., Nanjing, China). Soil pH data was measured in the field using an IQ-150 in situ pH meter (IQ Inc., Walnut, CA, USA). Soil organic carbon (SOC) was determined via wet oxidation using a total carbon analyzer (Shimadzu TOC-5000A, Kyoto, Japan), and total nitrogen (TN) was determined via dry combustion using an elemental analyzer (Vario EL III, Elementar, Langenselbold, Germany). Total phosphorus (TP) was determined after digestion with concentrated H_2_SO_4_–HClO_4_, followed by colorimetric analysis using the molybdenum–antimony (Mo–Sb) method with a UV–Vis spectrophotometer (Agilent, CA, USA). Total potassium (TK) was determined using flame photometry following H_2_SO_4_–HClO_4_ digestion. Available nitrogen (AN) was determined using the alkaline hydrolysis–diffusion method; available phosphorus (AP) was extracted with 0.5 mol L^−1^ NaHCO_3_ and determined using the Mo–Sb method; available potassium (AK) was determined using flame photometry [26]. Nitrate (NO_3_^−^–N) and ammonium (NH_4_^+^–N) were extracted with 2 mol L^−1^ KCl and determined using a continuous flow analyzer (SAN^++^8505, Skalar Analytical B.V., Breda, Netherlands). Soil microbial biomass carbon (MBC) and nitrogen (MBN) were determined using the chloroform fumigation–extraction method [27]. Dissolved organic carbon (DOC) and dissolved organic nitrogen (DON) were extracted with 0.5 mol L^−1^ K_2_SO_4_ and determined using a total organic carbon analyzer (Multi N/C 3100, Analytik Jena, Jena, Germany).

### 2.4. Measurements of Soil Enzyme Activity

Urease (UR) activity was determined using the sodium phenolate colorimetric method, protease (PRO) activity using ninhydrin colorimetry, and sucrase (SUC) activity using the 3,5-dinitrosalicylic acid (DNS) method [28]. Nitrate reductase (NR) activity was determined colorimetrically at 520 nm by measuring nitrite production, and nitrite reductase (NiR) activity was determined by monitoring nitrite consumption using the benzenesulfonic acid–acetic acid–1-naphthylamine method. Hydroxylamine reductase (HyR) activity was determined colorimetrically at 510 nm using the ammonium ferric sulfate–1,10-phenanthroline method. These enzymes were selected because they represent key steps in soil N transformation directly coupled to N_2_O production and consumption. UR and PRO convert organic N to NH_4_^+^, supplying substrates for nitrification. SUC reflects overall microbial activity and C turnover, influencing denitrifier metabolism. NR, NiR, and HyR are directly involved in denitrification and hydroxylamine reduction, a key nitrification intermediate.

### 2.5. Quantification of Soil Total Nitrification Rate, Denitrification Rate, and Related Gene Abundance

Total nitrification and denitrification rates were determined using the barometric process separation technique [29]. The abundances of nitrification-related genes (AOA–*amoA* and AOB–*amoA*) and denitrification-related genes (*nirK*, *nirS*, and *nosZ*) were determined via quantitative real-time PCR (qPCR) using SYBR Green chemistry on a CFX96 Real-Time PCR Detection System (Bio-Rad, Hercules, CA, USA). The quantitative PCR amplification reaction mixture consisted of 20 μL, comprising 10 μL SYBR Green, 0.2 μL Rox Dye II, 1 μL DNA template, 0.4 μL each of upstream and downstream primers (10 μM), and 8.0 μL sterile water. The experimental samples were sent to Shanghai Meiji Biomedical Technology Co., Ltd., Shanghai, China for bacterial DNA extraction, PCR amplification, gene library construction, and double-end sequencing.

### 2.6. Measurement of N_2_O Flux

Soil N_2_O fluxes were measured from January 2022 to December 2023, corresponding to the fifth and sixth years after a single biochar application. The soil N_2_O flux was determined using static headspace gas chromatography. A headspace incubation period of 30 min was employed, with a sampling container consisting of a 100 mL medical syringe equipped with a three-way valve. Approximately 100 mL of gas sample was collected while simultaneously recording the sampling time, chamber temperature, ambient air temperature, soil surface temperature, and temperature at a depth of 5 cm below the soil surface. Subsequently, samples were collected every 10 min; thus, each sampling chamber yielded four gas samples at 0, 10, 20, and 30 min after incubation. The collected gas samples were transported to the laboratory and injected into the injector of a gas chromatograph (HP 6890) for analysis of the target concentrations of the analytes. The annual cumulative flux was calculated by multiplying the average flux from two consecutive sampling periods by the interval between them and summing the results. The static chamber–gas chromatography method was used [30].

The N_2_O flux was calculated as follows:

$$N_{2}O=\rho \cdot \frac{V}{A}\cdot \frac{∆c}{∆t}=\rho \cdot H\cdot \frac{∆c}{∆t}$$

where N_2_O flux is the N_2_O emission flux (μg m^−2^ h^−1^); ρ is the gas density within the chamber (kg m^−3^); Δ*c* is the change in gas mixing ratio (ppbv) during chamber closure; Δ*t* is the time interval (h) over which the concentration change occurs; *A*, *V*, and *H* are the basal area (m^2^), volume (m^3^), and height (m) of the static chamber, respectively.

### 2.7. Statistical Analysis

Data were initially processed using Microsoft Excel 2024 (Microsoft Corp., Redmond, WA, USA). Pearson correlation analysis and one-way analysis of variance (ANOVA) were conducted using SPSS version 24.0 (IBM Corp., Armonk, NY, USA) with a significance level of α = 0.05. The hierarchical clustering heatmap was generated using R software (v.4.2.2) package “pheatmap” (v.1.0.12) through Hiplot Pro (https://hiplot.com.cn/). The primary factors influencing soil N_2_O emissions were identified and evaluated using structural equation modeling (SEM) in R software. All figures and tables were generated using Origin 2022 (OriginLab Corp., Northampton, MA, USA). Figure layouts were further refined and typeset using Adobe Illustrator 2024 (Adobe Inc., San Jose, CA, USA).

## 3. Results

### 3.1. Changes in Soil Temperature, Soil Water Content, and pH

Biochar application significantly modulated soil physicochemical properties for two consecutive growing seasons, most parameters exhibiting a clear dose-dependent trend (Table 1). Compared to the CK, ST showed a consistent but moderate increase, increasing by 0.47 °C in 2022 and 0.84 °C in 2023 under T5. SWC increased by 4.12 percentage points in 2022 and 4.97 percentage points in 2023 under T5. Furthermore, biochar application increased soil pH by 1.79 units in 2022 and 1.67 units in 2023 under T5. The monthly variations in these soil properties during the monitoring period are further detailed in Figure S1 in the Supplementary Materials. These results for 2022 and 2023, coupled with the highly significant differences (*p* < 0.01) observed for nearly all treatments, underscore the effects of biochar on soil thermal properties, water retention, and alkalinity.

### 3.2. Changes in Soil Nutrient and Carbon and Nitrogen Contents

**Figure 1:**
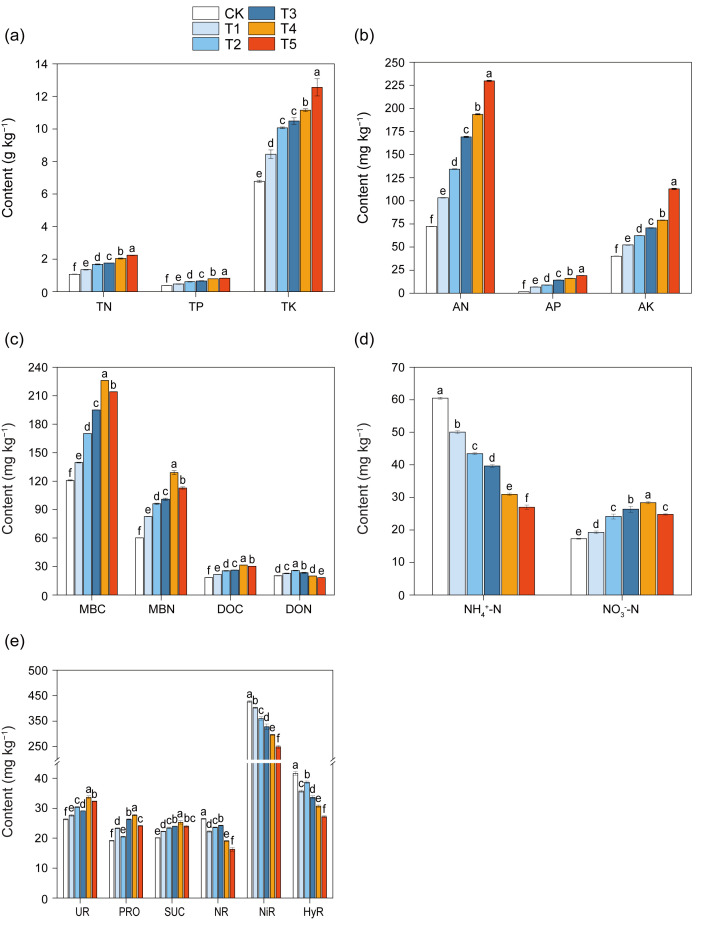
Dose-dependent responses of soil physicochemical properties, microbial biomass, and enzymatic activities to varying biochar application rates: (**a**) total potassium (TK), total nitrogen (TN), and total phosphorus (TP); (**b**) available nitrogen (AN), available potassium (AK), and available phosphorus (AP); (**c**) microbial biomass carbon (MBC), microbial biomass nitrogen (MBN), dissolved organic carbon (DOC), and dissolved organic nitrogen (DON); (**d**) ammonium nitrogen (NH_4_^+^-N) and nitrate nitrogen (NO_3_^−^-N); (**e**) urease (UR), protease (PRO), sucrase (SUC), nitrate reductase (NR), nitrite reductase (NiR), and hydroxylamine reductase (HyR). In all the panels, bars represent means ± standard deviation (SD) of three independent replicates (*n* = 3). Different lowercase letters indicate significant differences among treatments at *p* < 0.05.

TK, TN, TP, AN, AK, and AP generally increased with increasing biochar application rates (Figure 1a,b). MBC, MBN, and DOC showed unimodal responses; DON reached its maximum at T2 and declined thereafter (Figure 1c). Soil NH_4_^+^-N and NO_3_^−^-N concentrations responded differently to increasing biochar application rates (Figure 1d). NH_4_^+^-N content decreased consistently across treatments, corresponding to reductions of 17.3–55.4% compared to that in CK. In contrast, NO_3_^−^-N showed an increasing trend with increasing biochar application rate.

### 3.3. Changes in Soil Enzyme Activities

Soil enzyme activities responded differentially to biochar application (Figure 1e). UR, PRO, and SUC activities increased initially and peaked at T4, with increases of 27.8%, 44.7%, and 27.4%, respectively, compared to that in CK. In contrast, NR, NiR, and HyR decreased with increasing biochar rates, with reductions ranging from 35% to 42% compared to those in CK.

### 3.4. Changes in Soil N_2_O Emissions

**Figure 2:**
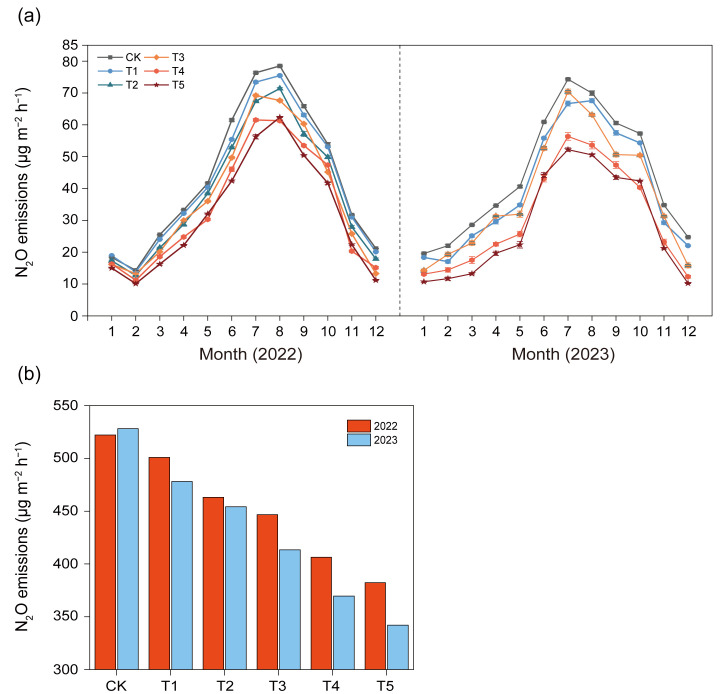
Changes in soil N_2_O emissions from January to December 2022 and 2023 (**a**) and their respective annual cumulative emissions (**b**).

Soil N_2_O emissions exhibited a seasonal pattern across all treatments, with a unimodal trajectory that peaked in mid-summer and declined thereafter in both 2022 and 2023 (Figure 2a). Biochar amendment significantly suppressed N_2_O emissions. In 2022, T4 achieved reduction rates of 27.25%, 21.90%, and 35.79% in May, August, and November, respectively, compared to the control, whereas T5 exhibited reduction rates ranging from 18.32% to 47.01% during the remaining months. In 2023, T4 showed reduction rates of 29.23% and 29.64% in June and September, respectively, while T5 maintained reduction rates ranging from 27.75% to 58.64% in the other months. In summary, while T4 showed the strongest suppression during specific emission months, higher application rates, particularly T5, generally maintained the lowest emission fluxes for the majority of the observation period.

Annually, cumulative N_2_O emissions decreased with increasing biochar application rate, indicating a dose–response relationship (Figure 2b). The mitigation effect was more pronounced in 2023 than in 2022, with reduction efficiencies ranging from 4.1% to 26.8% in 2022 and from 9.5% to 35.2% in 2023, based on the two monitored years. Statistical analysis identified soil temperature and moisture content as the primary drivers of N_2_O flux variability, and both were positively correlated with emissions in both years (*p* < 0.01; Supplementary Materials, Figure S2). The influence of pH was not significant in 2022, but a significant negative correlation was observed in 2023 (*p* < 0.05), indicating that biochar-induced alkalization played a more significant role in emission suppression in 2023 compared to 2022. Overall, these results demonstrate that biochar application effectively reduced soil N_2_O emissions, with a higher mitigation efficiency observed in the second monitored year (2023) under strong environmental regulation.

### 3.5. Changes in Nitrification and Denitrification

**Figure 3:**
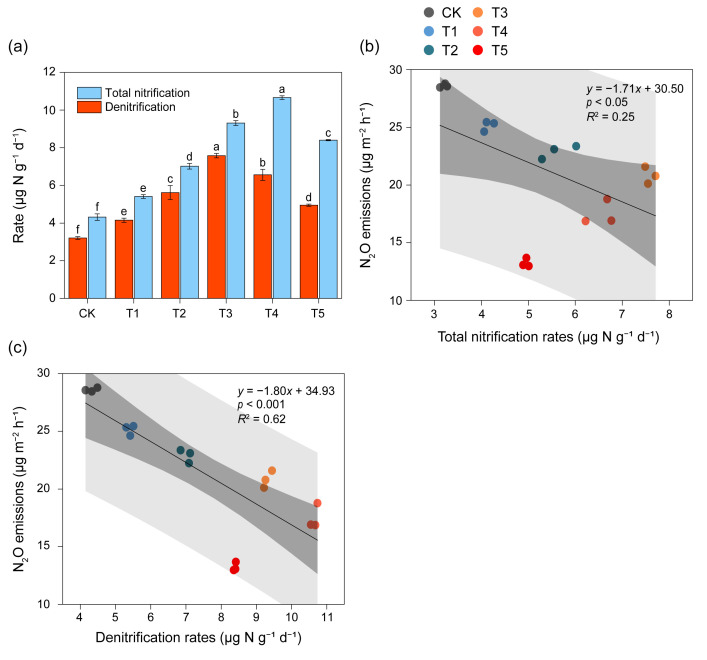
Impact of biochar application rates on nitrogen transformation processes and their relationships with N_2_O emissions. (**a**) Responses of total nitrification rate (TNR) and denitrification rate (DNR) to different biochar application rates. Error bars indicate S. D (*n* = 3), and different letters denote significant differences among treatments (*p* < 0.05). (**b**,**c**) Linear relationships between N_2_O emissions and TNR (**b**) and DNR (**c**). Solid lines represent linear fits, and the shaded areas denote 95% confidence intervals.

Both total nitrification (TNR) and denitrification (DNR) rates exhibited unimodal responses to increasing biochar application, with peak values observed in T3 and T4 treatments, respectively (Figure 3a). TNR reached a maximum of 7.58 μg N g^−1^ d^−1^ in T3, that is, a 2.36-fold increase compared to that in CK. Similarly, DNR reached its highest value in T4 (10.66 μg N g^−1^ d^−1^), that is, a 2.46-fold increase compared to that in the control. Linear regression analysis revealed significant negative relationships between N_2_O emissions and both TNR and DNR (Figure 3b,c). Specifically, N_2_O emissions negatively correlated with TNR (*R*^2^ = 0.25, *p* < 0.05) and exhibited a stronger negative correlation with DNR (*R*^2^ = 0.62, *p* < 0.001). These results suggest that denitrification may play a more important role than nitrification in regulating net N_2_O emissions under biochar amendment.

### 3.6. Changes in Nitrogen-Cycle-Related Functional Genes

**Figure 4:**
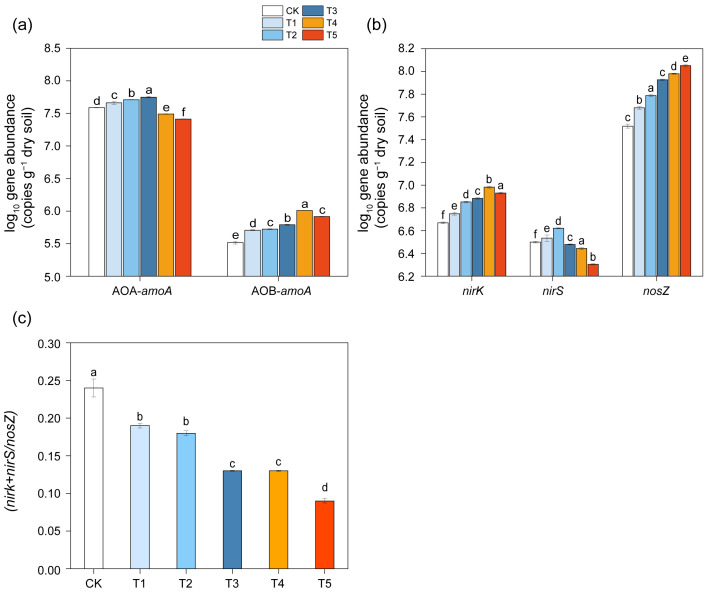
Dose-dependent responses of soil nitrogen cycling functional gene abundances and potential denitrification balance to varying biochar application rates: (**a**) nitrification-related genes (AOA-*amoA* and AOB-*amoA*), (**b**) denitrification-related genes (*nirK*, *nirS*, and *nosZ*), and (**c**) (*nirK* + *nirS*)/*nosZ* ratio. Gene abundance values in panels (**a**,**b**) are presented on a log_10_ scale (copies g^−1^ dry soil). In all the panels, bars represent means ± standard deviation (SD) of three independent replicates (*n* = 3). Different lowercase letters indicate significant differences among treatments at *p* < 0.05.

The abundance of AOA-*amoA* exhibited a unimodal response to biochar addition, reaching its maximum at moderate application rates before declining thereafter. In contrast, AOB-*amoA* abundance displayed a dose-dependent increase across the gradient (Figure 4a). Compared to that in the CK, AOA-*amoA* abundance was significantly suppressed under high-dosage treatments (T4 and T5). Notably, AOA-*amoA* maintained numerical predominance over AOB-*amoA* across all treatments, underscoring its pivotal role in ammonia oxidation within the acidic soil matrix investigated in this study. Compared with that in the CK, the abundances of *nirS* followed a non-monotonic trend, characterized by an initial increase followed by a subsequent decline at elevated biochar rates (Figure 4b). Conversely, *nosZ* and *nirK* abundance showed a robust monotonic increase, reaching the maximum value in T5 and T4 treatments compared to that in CK treatment. (*nirK* + *nirS*)/*nosZ* ratio underwent a significant dose-dependent reduction, suggesting a potential shift toward complete denitrification (Figure 4c).

### 3.7. Correlation Analysis of Physicochemical Properties, Enzyme Activities, and Nitrogen-Cycle-Related Functional Genes

**Figure 5:**
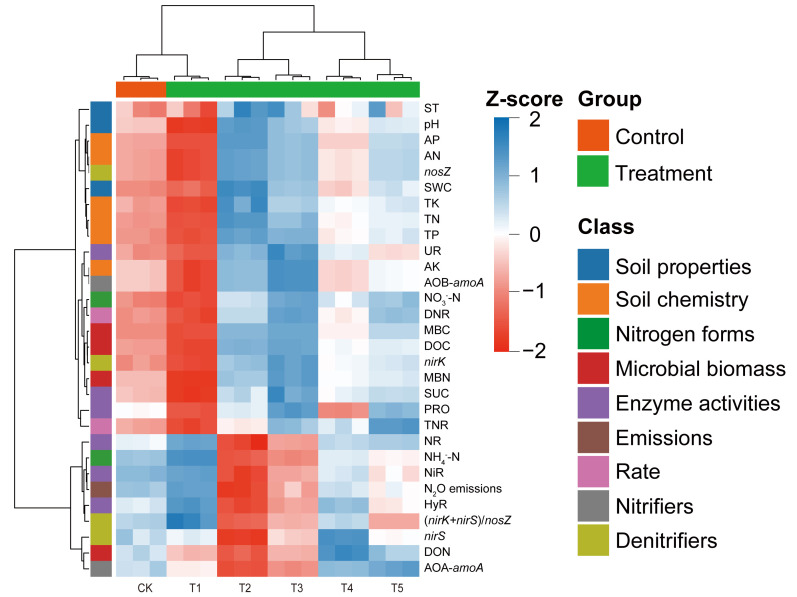
Hierarchical clustering heatmap showing the coordinate shifts in soil physicochemical properties, enzyme activities, and nitrogen cycling functional genes across treatments. The original data were standardized using Z-scores to a range of −2.0 (red, below-average intensity) to 2.0 (blue, above-average intensity). Hierarchical clustering of both parameters and treatments was executed using the Ward.D2 algorithm based on Euclidean distances to identify structural similarities and divergent treatment responses. The dendrograms at the top and left represent the clustering relationships among treatments and variables, respectively.

Hierarchical clustering analysis and the associated heatmap further elucidated the divergent responses of soil physicochemical properties, enzymatic activities, and nitrogen cycle indices to biochar amendment (Figure 5). The treatments were bifurcated into two primary clusters: the CK and T1 formed a cohesive group, whereas the higher-dosage biochar treatments (T4 and T5) were distinctly segregated. This clustering hierarchy underscores a dose-dependent shift in the soil microenvironment induced by substantial biochar input. Specifically, high-dosage treatments (T4 and T5) were characterized by a synergistic enrichment of soil moisture, total nutrients (TN, TP, and TK), and inorganic N dominated by nitrate (NO_3_^−^-N), as evidenced by their elevated standardized Z-scores for these specific parameters. Conversely, these treatments exhibited depletion in ammonium (NH_4_^+^-N) and DON. These coordinate shifts in soil properties and microbial functional traits suggest that biochar-driven alterations in N-cycling pathways are the primary drivers behind the observed variations in N_2_O emission patterns.

### 3.8. SEM Model and Standardised Impact Values

**Figure 6:**
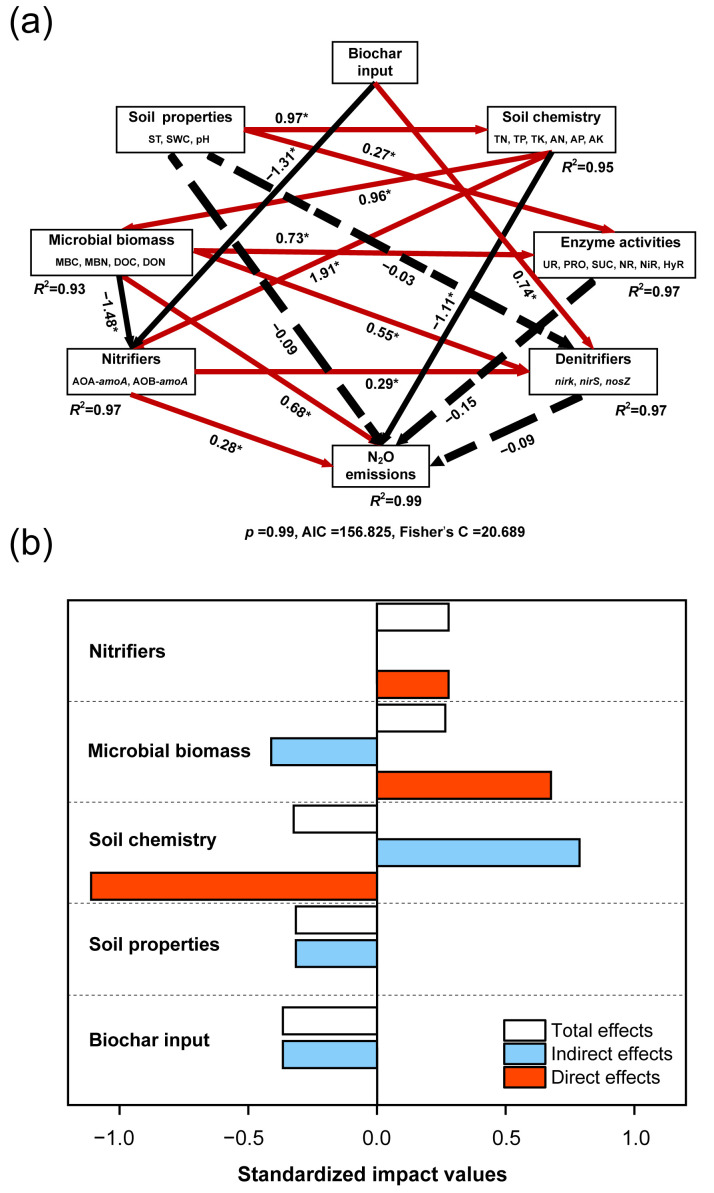
SEM model (**a**) and standardized impact values (**b**). Path diagram illustrating the direct and indirect effects of biochar input on soil properties, microbial functions, and N_2_O emissions. Numbers on arrows are standardised path coefficients. Significant paths are marked with asterisks *. Solid red arrows indicate significant positive effects; solid black arrows indicate significant negative effects; dashed black arrows denote non-significant effects. The coefficient of determination (*R*^2^) for each response variable is adjacent to its box.

SEM model illustrated the direct and indirect pathways linking soil physicochemical properties, microbial biomass, enzyme activity, nitrification and denitrification processes, and N_2_O emissions (Figure 6). The model showed an acceptable fit (AIC = 156.825, Fisher’s C = 20.689, *p* > 0.05). ST, SWC, and pH had a significant positive effect on soil chemical properties (path coefficient = 0.97, *p* < 0.05). Improved soil chemical properties subsequently promoted microbial biomass (path coefficient = 0.96, *p* < 0.05), which further stimulated soil enzyme activity (path coefficient = 0.73, *p* < 0.05). Biochar input showed a significant negative effect on the gene abundances of AOA-*amoA* and AOB-*amoA* (path coefficient = −1.31, *p* < 0.05); however, nitrifiers remained a significant positive driver of N_2_O emissions (path coefficient = 0.28, *p* < 0.05). In contrast, soil chemical properties exhibited a strong direct negative effect on N_2_O emissions (path coefficient = −1.11, *p* < 0.05). Overall, the model explained 99% of the variance in N_2_O emissions, indicating that soil physicochemical and microbial variables accounted for most of the observed variation.

These results suggest that the variation in N_2_O emissions was controlled by the balance between two interacting pathways. On the one hand, biochar directly suppressed the abundance of nitrifying microorganisms, which may reduce nitrification-derived N_2_O production. On the other hand, biochar indirectly enhanced microbial biomass and enzyme activity by improving soil properties, potentially stimulating microbial groups involved in N_2_O reduction, particularly those harboring the *nosZ*.

## 4. Discussion

### 4.1. Effect of Biochar Application on Soil N_2_O Emissions in Eucalyptus Plantations

This study reveals that amendment with *Eucalyptus* branch-derived biochar induced a consistent, dose-dependent attenuation of both soil N_2_O fluxes and cumulative emissions. This mitigation effect is consistent with global meta-analyses on significant N_2_O reductions following biochar incorporation across diverse ecosystems [31]. Biochar-induced suppression appears to arise largely from improvements in soil physicochemical properties. Biochar amendment significantly elevated soil alkalinity, microbial biomass carbon and nitrogen, and nutrient content. Significant negative correlations (*p* < 0.01) between N_2_O emissions and these soil parameters further indicate that biochar-induced environmental improvements, particularly the substantial reduction in soil acidity (with pH increasing by up 1.79 units), effectively suppress the substrate-driven potential for N_2_O generation [32]. However, it is necessary to consider the potential confounding effects of soil physical changes. Table 1 illustrates that high biochar application increased soil temperature by up to 0.84 °C. While this minor warming theoretically stimulates microbial metabolism and baseline N_2_O production, it failed to disrupt the overall emission reduction trend. Potential temperature-driven stimulation was ultimately outweighed by the primary physicochemical and biological mechanisms of biochar, namely soil alkalization and the enrichment of *nosZ* genes.

Furthermore, biochar application significantly influenced N transformation rates. In the T4 treatment, a significant decrease in NH_4_^+^-N content was accompanied by a peak in NO_3_^−^-N concentration. This pattern suggests that biochar facilitates nitrification and potentially promotes N immobilization, thereby reducing the mineral N pool available for N_2_O generation, reshaping the substrate availability for subsequent microbial pathways. Although total nitrification and denitrification rates were enhanced, the strong negative correlation (*p* < 0.01) between denitrification rates and N_2_O emissions suggests that denitrification plays a more important role than nitrification in regulating net N_2_O emissions under biochar amendment, potentially leading to a more complete denitrification process [33,34,35]. The coexistence of enhanced nitrification and reduced net N_2_O emissions is primarily driven by the substantial enrichment of *nosZ* gene abundance, which ensures rapid reduction of intermediately produced N_2_O to N_2_. Additionally, biochar-induced improvements in soil aeration and NH_4_^+^-N adsorption further modulate the microenvironment, collectively reconciling this apparent paradox. This optimization may effectively redirect a greater proportion of reactive N toward the production of benign N_2_ gas. Additionally, this mitigation pattern is conceptually consistent with the established paradigm of biochar acting as an extracellular electron shuttle, facilitating inter-microbial electron transfer that sustains the energetically intensive reduction of N_2_O catalyzed by the *nosZ* enzyme [36].

In the present study, the sustained mitigation observed underscores a long-term microbial optimization process, potentially involving the remodeling of key metabolic pathways directly involved in N_2_O production [37]. SEM model analysis further suggests that this mitigation effect is controlled by a bifurcated regulatory framework involving direct improvement in soil chemistry and indirect modulation of microbial functional communities.

### 4.2. Effect of Biochar on the Abundance of Soil N-Cycle Functional Genes

Biochar application significantly influenced the abundance of key microbial functional genes involved in soil N cycle, thereby regulating nitrification processes and net N_2_O emissions [38]. The contrasting responses of their respective *amoA* genes point toward a potential niche differentiation between AOA and AOB, suggesting that these two nitrifying guilds possess distinct adaptive thresholds within the biochar-modified microenvironment [39,40]. Biochar amendment significantly enhanced AOB-*amoA* abundance in a dose-dependent manner, whereas AOA-*amoA* abundance exhibited a unimodal response in *Eucalyptus* plantations, peaking at intermediate application rates before declining [41]. This ecological differentiation likely originates from biochar-induced changes in soil microenvironments, where elevated pH and microbial biomass carbon and nitrogen align more closely with the physiological requirements of copiotrophic AOB [42]. Beyond pH and substrate preferences, the marked decline in AOA at high biochar dosages may also involve direct toxicity from biochar-derived substances [43]. Residual volatile organic compounds and excessive salts accumulated locally could induce environmental stress that disproportionately inhibits sensitive oligotrophic archaea [44].

Despite the positive response of AOB, the absolute abundance of AOA-*amoA* consistently exceeded that of AOB-*amoA* throughout the study period. This pattern highlights the fundamental role of oligotrophic AOA in maintaining baseline nitrification in acidic forest soils [45,46,47]. For denitrification, biochar amendment significantly increased *nosZ* gene abundance, with a 3.4-fold increase in T5 treatment and concurrent reduction in (*nirK* + *nirS*)/*nosZ* ratio. This functional shift, accompanied by rising NO_3_^−^-N concentrations, signals a transition toward a more complete denitrification pathway that favours the reduction of N_2_O to N_2_ [48,49]. Similarly, [50] demonstrated that rice straw biochar significantly boosted the abundance of *nosZ* and *nirK* genes in acidic soils, indicating an enhanced microbial genetic potential for N_2_O consumption.

This transition appears to be driven by two interconnected mechanisms. First, biochar improves soil physical structure and aeration, which help to create micro-aerobic conditions that stimulate *nosZ*-harboring microorganisms and reduce the advantage of anaerobic denitrifiers [51]. Second, biochar likely acts as an electron mediator, enhancing the transfer efficiency required for the energy-intensive reduction step catalyzed by the *nosZ* enzyme [52]. Collectively, these coordinated functional gene responses indicate that biochar can directionally steer microbial community function pathways that favor the mitigation of N_2_O emissions, ultimately mitigating net GHG emissions in subtropical plantations [53].

### 4.3. Limitations and Future Prospects

While this study provides valuable insights, several limitations remain. First, a notable limitation of the present study is that it did not evaluate the role of arbuscular mycorrhizal (AM) fungi. Although AM fungi are widely recognized to influence soil nitrogen cycling, nitrification, denitrification, and nitrogen leaching, their responses to biochar application and potential interactions with N-cycling microbes were not assessed here, leaving a gap in fully understanding the microbial mechanisms of N_2_O mitigation. Second, this study did not perform high-throughput sequencing to analyze the composition and diversity of *narG*, *nirK*, *nirS*, and *nosZ* denitrifying bacterial communities. Therefore, the impact of biochar on soil denitrifying bacterial community composition and diversity, as well as their main influencing factors, remains unclear within the scope of this study. Third, despite observing significant mitigation effects six years after a single biochar application, this study lacks continuous interannual monitoring of N_2_O fluxes and soil properties throughout the entire experimental period. Hence, the interannual dynamic changes and long-term temporal legacy differences in soil N_2_O emissions need to be further elucidated through extended, multi-site field experiments. Future research should establish long-term continuous monitoring to better guide the practical application of biochar in subtropical forestry.

## 5. Conclusions

Biochar-amended soil decreased N_2_O flux and cumulative N_2_O emission compared to non-amended soils. The high adsorption capacity for NH_4_^+^-N and N_2_O and the enhanced soil aeration in biochar-amended soil may explain the reduction in N_2_O emissions after six years of application. Biochar amendment reconstructs a soil microenvironment that is less favorable for N_2_O generation through synergistic improvements in pH, enzyme activity, microbial biomass carbon and nitrogen, dissolved organic carbon and nitrogen, and nutrient content. This regulatory framework operates through the direct improvement in soil physicochemical properties and the closely associated adaptive responses of microbial functional communities that correlate with the mitigated N_2_O pathways. These findings demonstrate the applicability of biochar amendment to this subtropical plantation system, providing localized evidence for regional greenhouse gas mitigation and facilitating the sustainable valorization of forestry wastes.

## Figures and Tables

**Table 1:** Effects of biochar application on soil basic properties (ST, SWC, and pH) in two growing seasons.

Soil Properties	Group	Mean ± SD (2022)	Δ*T* (°C)	Mean ± SD (2023)	Δ*T* (°C)
Soil temperature (°C)	CK	18.60 ± 0.03 ^f^		18.59 ± 0.04 ^e^	
	T1	18.71 ± 0.02 ^e^	+0.11	18.87 ± 0.10 ^d^	+0.28
	T2	18.81 ± 0.02 ^d^	+0.21	19.04 ± 0.03 ^c^	+0.44
	T3	18.87 ± 0.02 ^c^	+ 0.27	19.12 ± 0.03 ^bc^	+ 0.52
	T4	18.94 ± 0.01 ^b^	+0.34	19.23 ± 0.01 ^b^	+0.64
	T5	19.07 ± 0.02 ^a^	+0.47	19.44 ± 0.16 ^a^	+0.84
Soil water content (%)	CK	18.76 ± 0.08 ^f^		20.28 ± 0.03 ^f^	
	T1	19.27 ± 0.07 ^e^	+0.51	21.27 ± 0.08 ^e^	+0.99
	T2	20.46 ± 0.03 ^d^	+1.69	21.88 ± 0.11 ^d^	+1.61
	T3	21.07 ± 0.03 ^c^	+2.30	23.22 ± 0.06 ^b^	+2.94
	T4	22.05 ± 0.10 ^b^	+3.28	24.12 ± 0.04 ^c^	+3.84
	T5	22.89 ± 0.06 ^a^	+4.12	25.25 ± 0.08 ^a^	+4.97
pH	CK	5.40 ± 0.00 ^f^		5.39 ± 0.00 ^f^	
	T1	6.17 ± 0.00 ^e^	+0.77	6.13 ± 0.01 ^e^	+0.74
	T2	6.38 ± 0.00 ^d^	+0.98	6.27 ± 0.00 ^d^	+0.88
	T3	6.70 ± 0.01 ^c^	+1.30	6.57 ± 0.02 ^c^	+1.19
	T4	6.89 ± 0.01 ^b^	+1.49	6.74 ± 0.02 ^b^	+1.35
	T5	7.19 ± 0.01 ^a^	+1.79	7.05 ± 0.02 ^a^	+1.67

Values are presented as the annual mean ± standard deviation (SD, *n* = 3). The temperature increase relative to the control (CK) within the same year is shown as ∆*T* (°C). Different lowercase letters (a, b, c, …) indicate statistically significant differences among treatments based on a one-way ANOVA (*p* < 0.05).

## Data Availability

The data supporting the findings of this study are available from the corresponding authors upon reasonable request.

## Supplementary Materials

The following supporting information can be downloaded at https://www.mdpi.com/article/10.3390/microorganisms14071519/s1, Table S1: Physicochemical properties of the soil. Table S2: Basic properties of the biochar. Figure S1: Monthly variations in soil temperature (ST), soil water content (SWC), and soil pH under different treatments in 2022 and 2023: (a) January; (b) February; (c) March; (d) April; (e) May; (f) June; (g) July; (h) August; (i) September; (j) October; (k) November; (l) December. Different lowercase letters indicate significant differences among treatments at *p* < 0.05. Figure S2: Relationship between N_2_O emissions and ST, SWC, and pH in 2022 and 2023.
