# Supplementary material for: Six-Year Biochar Experiment Reduces Soil N2O Emissions in Eucalyptus Plantations: Associations with Microbial N-Cycle Genes
Source: Microorganisms. 2026 Jul 12;14(7):1519. doi: 10.3390/microorganisms14071519 (PMC13414158; doi:10.3390/microorganisms14071519)
Supplement: Supplementary file 1 [file microorganisms-14-01519-s001.zip › microorganisms-4381512-supplementary.pdf]

Table S1 Physicochemical properties of the soil.

physicochemical properties	Value	Unit
pH	5.31	
Cation exchange capacity (CEC)	6.95	$cmol·kg^{-1}$
Electrical conductivity (EC)	31.69	$μS·cm^{-1}$
Soil water content (SWC)	19.84	%
Bulk density (BD)	1.72	$g·cm^{-3}$
Soil organic carbon (SOC)	5.53	$g·kg^{-1}$
Total nitrogen (TN)	0.94	$g·kg^{-1}$
Total phosphorus (TP)	0.24	$g·kg^{-1}$
Total potassium (TK)	4.8	$g·kg^{-1}$
Available nitrogen (AN)	55.21	$mg·kg^{-1}$
Available phosphorus (AP)	1.29	$g·kg^{-1}$
Available potassium (AK)	29.22	$mg·kg^{-1}$

Table S2 Basic properties of the biochar.

physicochemical properties	Value	Unit
pH	9.52	
Specific surface area (SSA)	43.21	$m^2·g^{-1}$
Electrical conductivity (EC)	692.82	$μS·cm^{-1}$
Cation exchange capacity (CEC)	51.48	$cmol·kg^{-1}$
Ash Content (Ash)	41.05	%
Carbon content （C）	55.86	%
Hydrogen content （H）	3.13	%
Oxygen content (O)	23.70	%
Nitrogen content (N)	1.36	%
Phosphorus Content (P)	5.46	$mg·kg^{-1}$
Potassium content (K)	32.48	$mg·kg^{-1}$
Available phosphorus (AP)	102.28	$g·kg^{-1}$

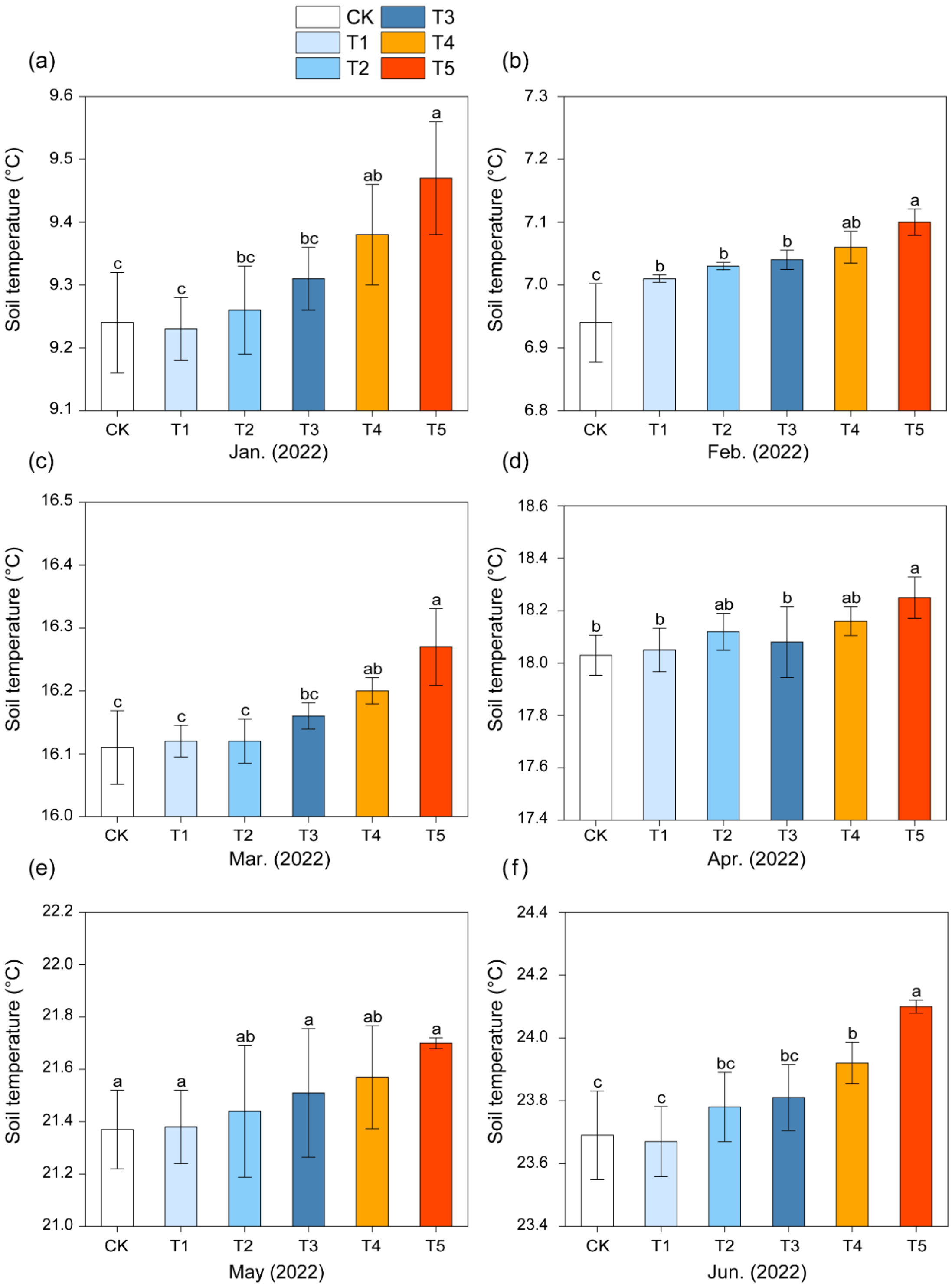

CK
T1
T2
T3
T4
T5
(a)
Soil temperature (°C)
Jan. (2022)
(b)
Feb. (2022)
(c)
Mar. (2022)
(d)
Apr. (2022)
(e)
May (2022)
(f)
Jun. (2022)

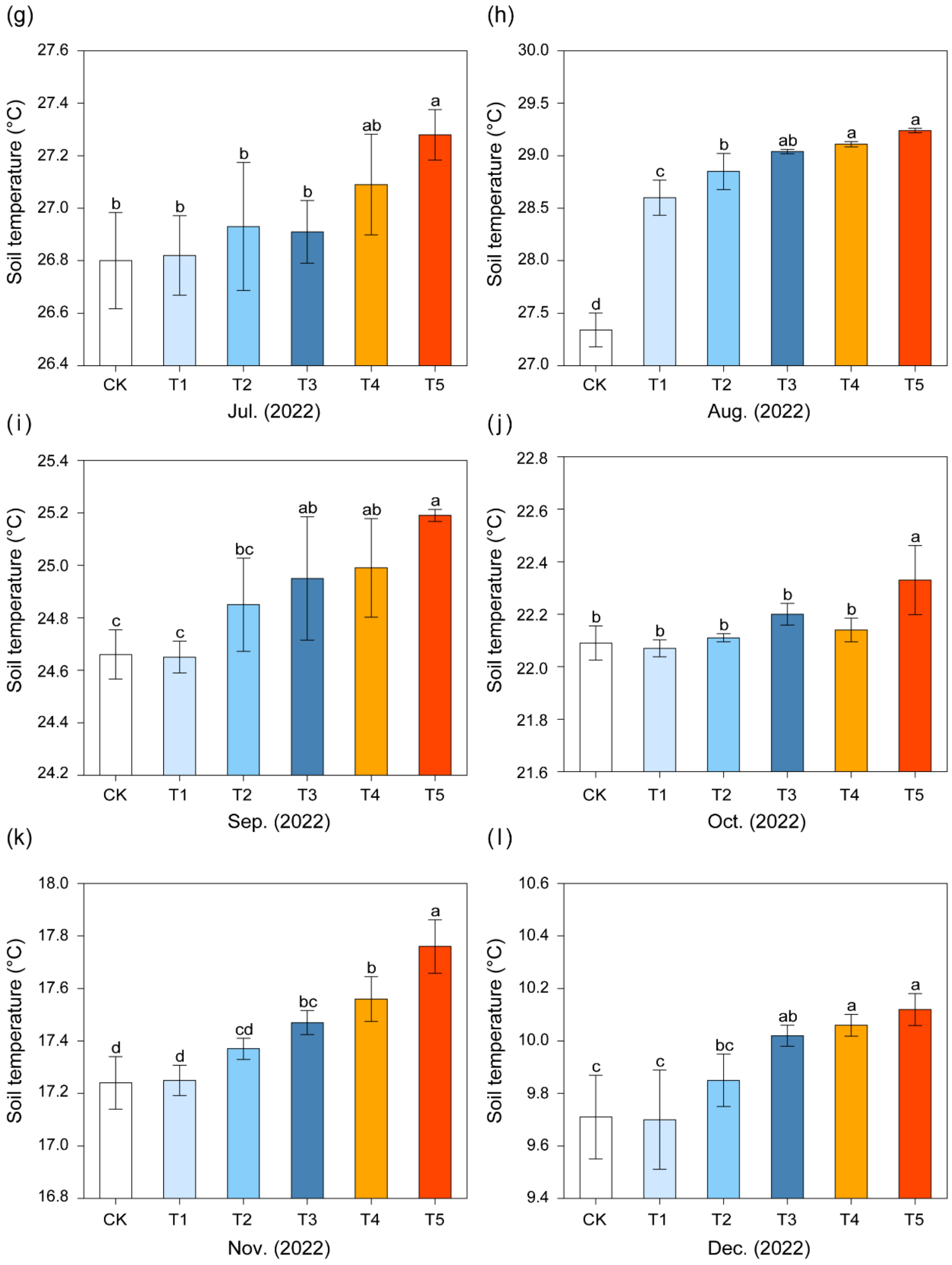

(g)
Soil temperature (°C)
27.6
27.4
27.2
27.0
26.8
26.6
26.4
b b b b ab a
CK T1 T2 T3 T4 T5
Jul. (2022)
(h)
Soil temperature (°C)
30.0
29.5
29.0
28.5
28.0
27.5
27.0
d c b ab a a
CK T1 T2 T3 T4 T5
Aug. (2022)
(i)
Soil temperature (°C)
25.4
25.2
25.0
24.8
24.6
24.4
24.2
c c bc ab ab a
CK T1 T2 T3 T4 T5
Sep. (2022)
(j)
Soil temperature (°C)
22.8
22.6
22.4
22.2
22.0
21.8
21.6
b b b b b a
CK T1 T2 T3 T4 T5
Oct. (2022)
(k)
Soil temperature (°C)
18.0
17.8
17.6
17.4
17.2
17.0
16.8
d d cd bc b a
CK T1 T2 T3 T4 T5
Nov. (2022)
(l)
Soil temperature (°C)
10.6
10.4
10.2
10.0
9.8
9.6
9.4
c c bc ab a a
CK T1 T2 T3 T4 T5
Dec. (2022)

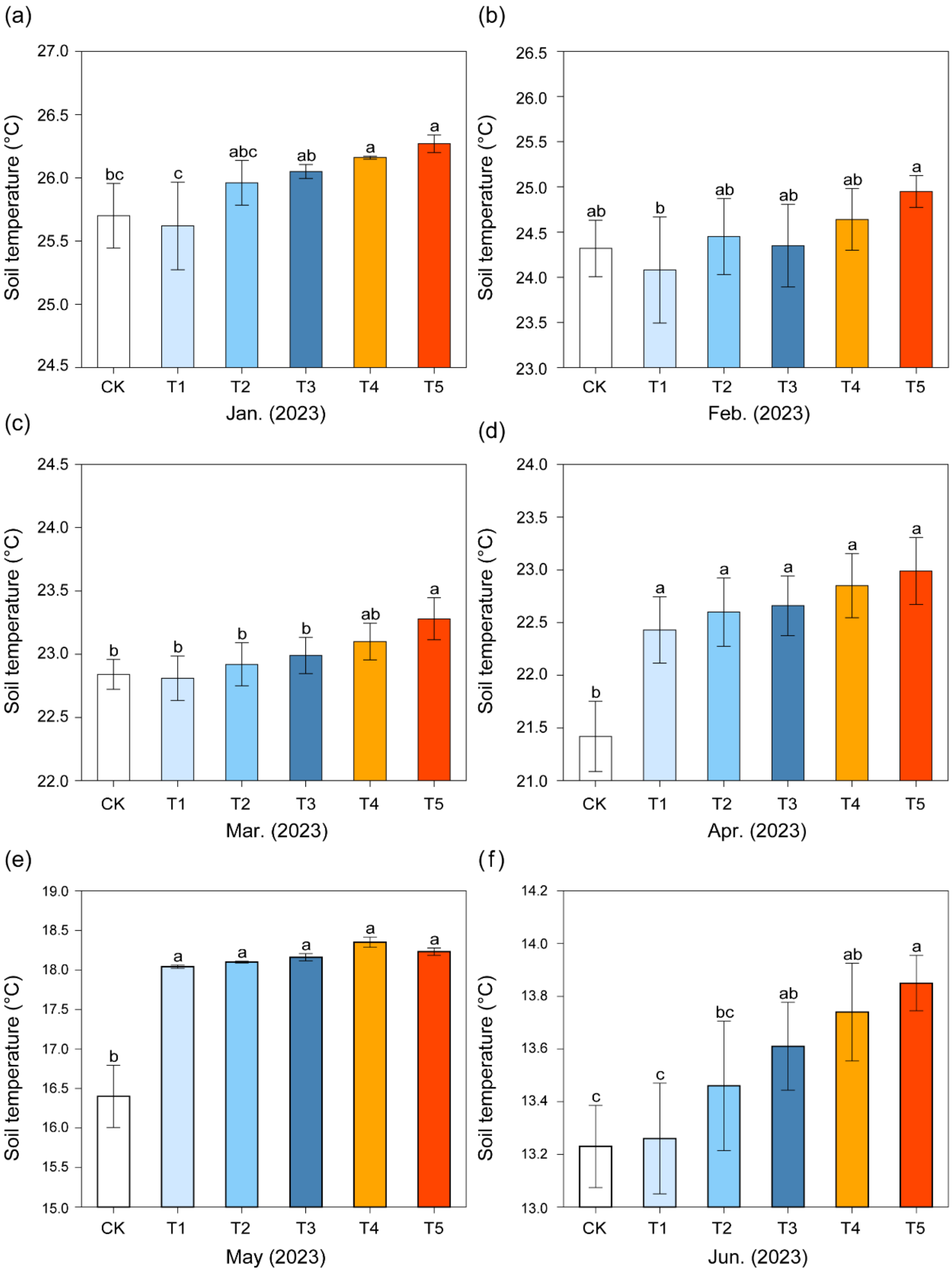

(a)
(b)
(c)
(d)
(e)
(f)
Soil temperature (°C)
CK
T1
T2
T3
T4
T5
Jan. (2023)
Feb. (2023)
Mar. (2023)
Apr. (2023)
May (2023)
Jun. (2023)
bc
c
abc
ab
a
a
ab
b
ab
ab
ab
a
b
b
b
b
ab
a
b
a
a
a
a
a
b
a
a
a
a
a
c
c
bc
ab
ab
a

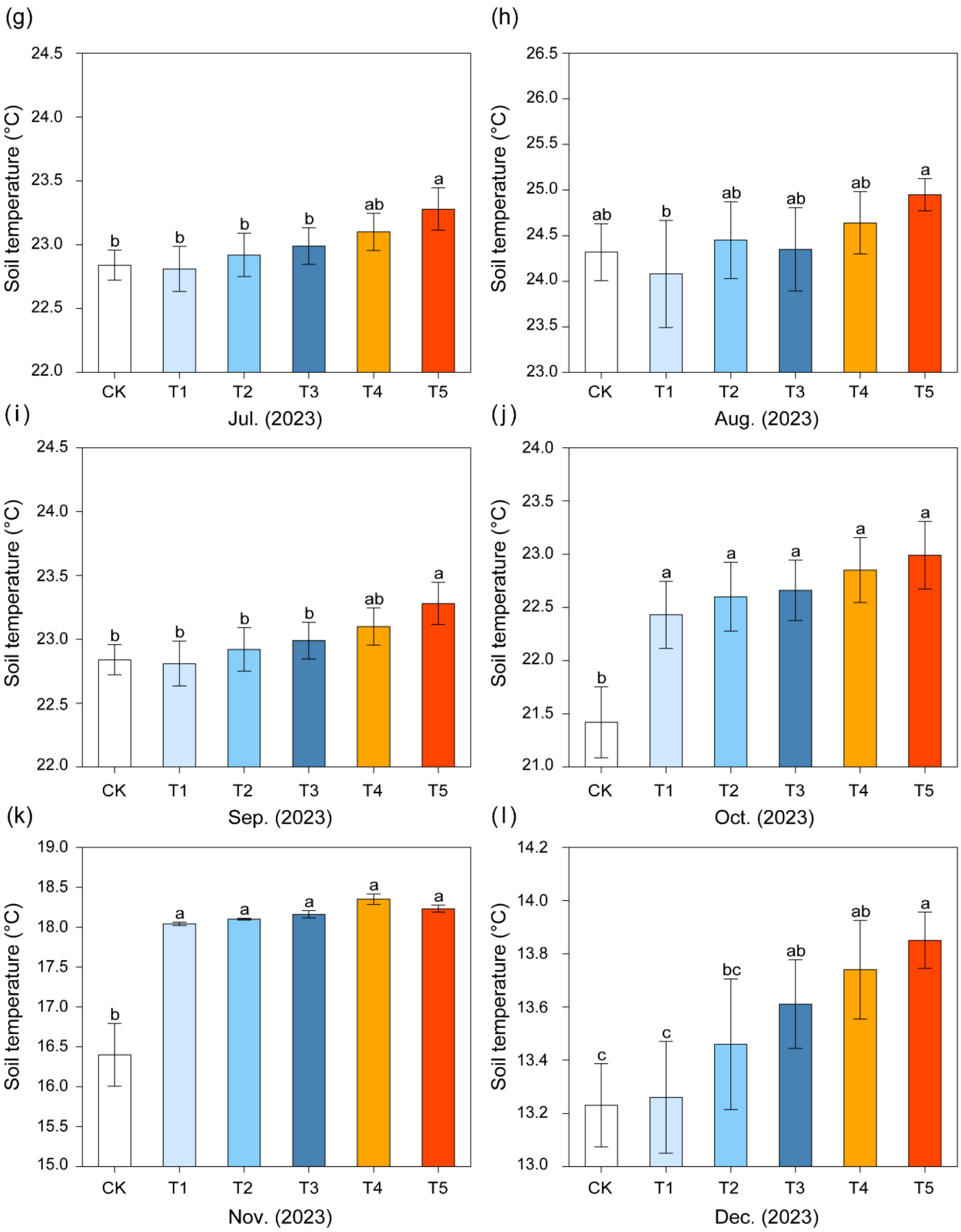
(g)
Soil temperature (°C)
24.5
24.0
23.5
23.0
22.5
22.0
b b b b ab a
CK T1 T2 T3 T4 T5
Jul. (2023)
(h)
Soil temperature (°C)
26.5
26.0
25.5
25.0
24.5
24.0
23.5
23.0
ab b ab ab ab a
CK T1 T2 T3 T4 T5
Aug. (2023)
(i)
Soil temperature (°C)
24.5
24.0
23.5
23.0
22.5
22.0
b b b b ab a
CK T1 T2 T3 T4 T5
Sep. (2023)
(j)
Soil temperature (°C)
24.0
23.5
23.0
22.5
22.0
21.5
21.0
b a a a a a
CK T1 T2 T3 T4 T5
Oct. (2023)
(k)
Soil temperature (°C)
19.0
18.5
18.0
17.5
17.0
16.5
16.0
15.5
15.0
b a a a a a
CK T1 T2 T3 T4 T5
Nov. (2023)
(l)
Soil temperature (°C)
14.2
14.0
13.8
13.6
13.4
13.2
13.0
c c bc ab ab a
CK T1 T2 T3 T4 T5
Dec. (2023)

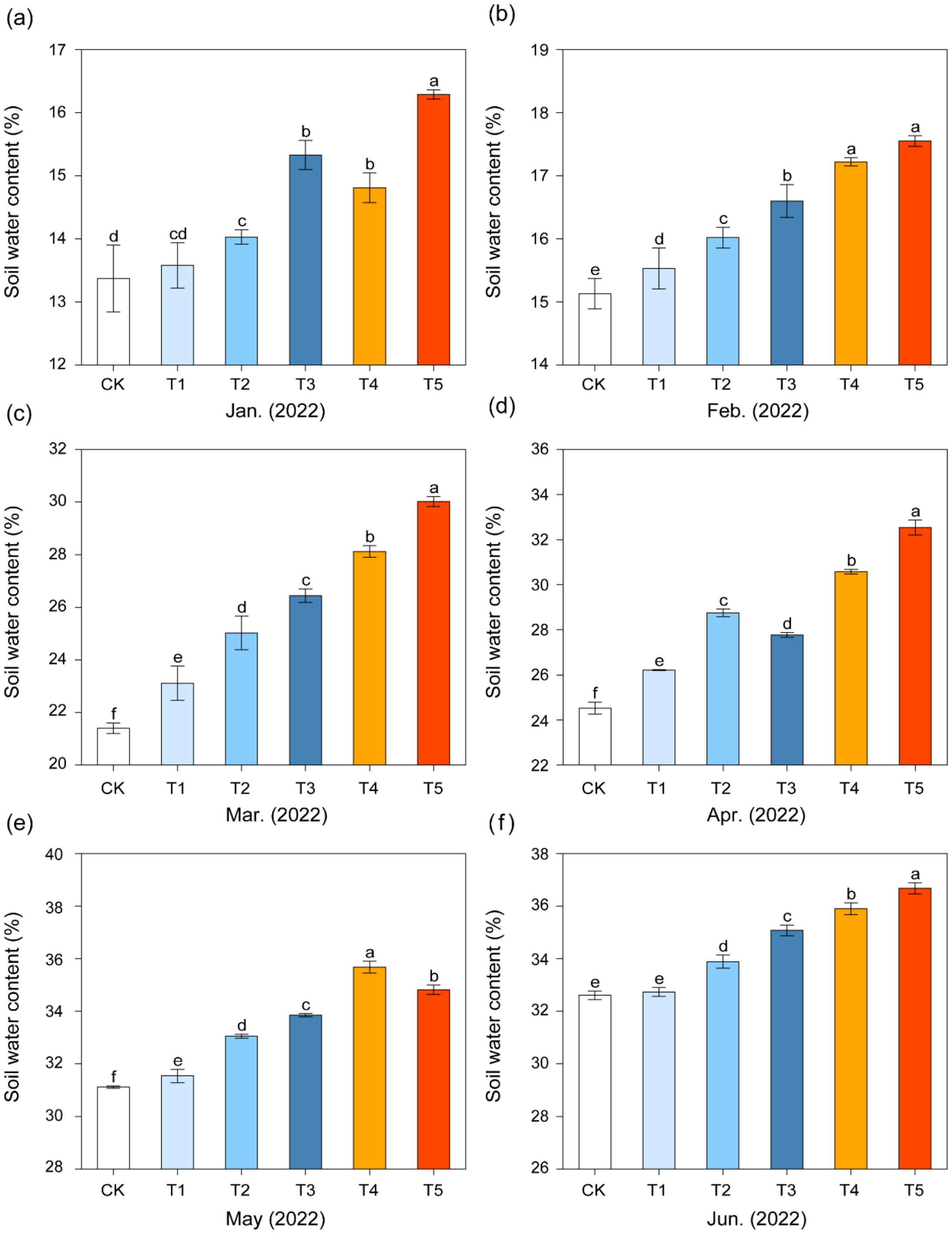

(a)
Soil water content (%)
17
16
15
14
13
12
d
cd
c
b
b
a
CK
T1
T2
T3
T4
T5
Jan. (2022)
(b)
Soil water content (%)
19
18
17
16
15
14
e
d
c
b
a
a
CK
T1
T2
T3
T4
T5
Feb. (2022)
(c)
Soil water content (%)
32
30
28
26
24
22
20
f
e
d
c
b
a
CK
T1
T2
T3
T4
T5
Mar. (2022)
(d)
Soil water content (%)
36
34
32
30
28
26
24
22
f
e
c
d
b
a
CK
T1
T2
T3
T4
T5
Apr. (2022)
(e)
Soil water content (%)
40
38
36
34
32
30
28
f
e
d
c
a
b
CK
T1
T2
T3
T4
T5
May (2022)
(f)
Soil water content (%)
38
36
34
32
30
28
26
e
e
d
c
b
a
CK
T1
T2
T3
T4
T5
Jun. (2022)

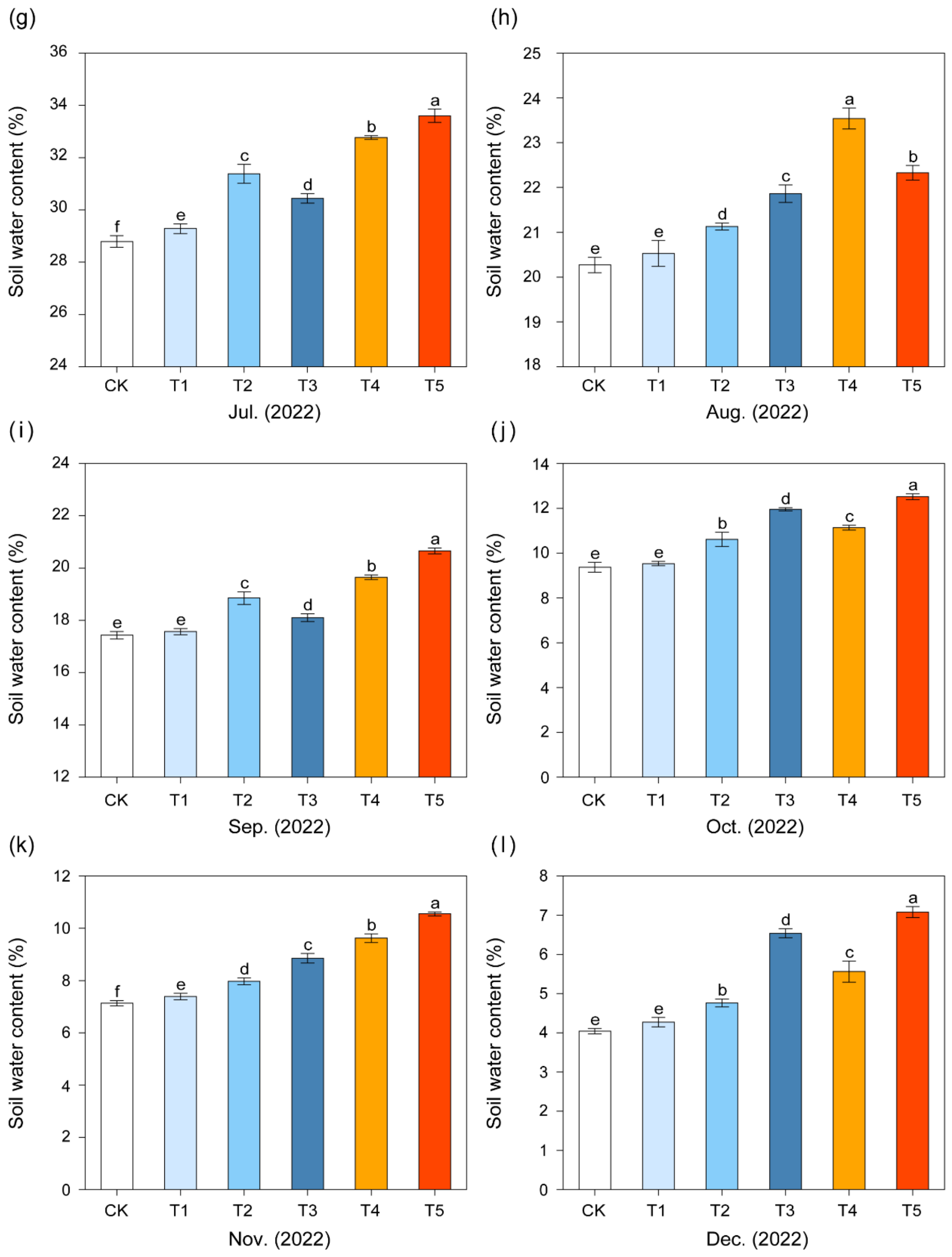

(g)
(h)
(i)
(j)
(k)
(l)
Soil water content (%)
CK
T1
T2
T3
T4
T5
Jul. (2022)
Aug. (2022)
Sep. (2022)
Oct. (2022)
Nov. (2022)
Dec. (2022)

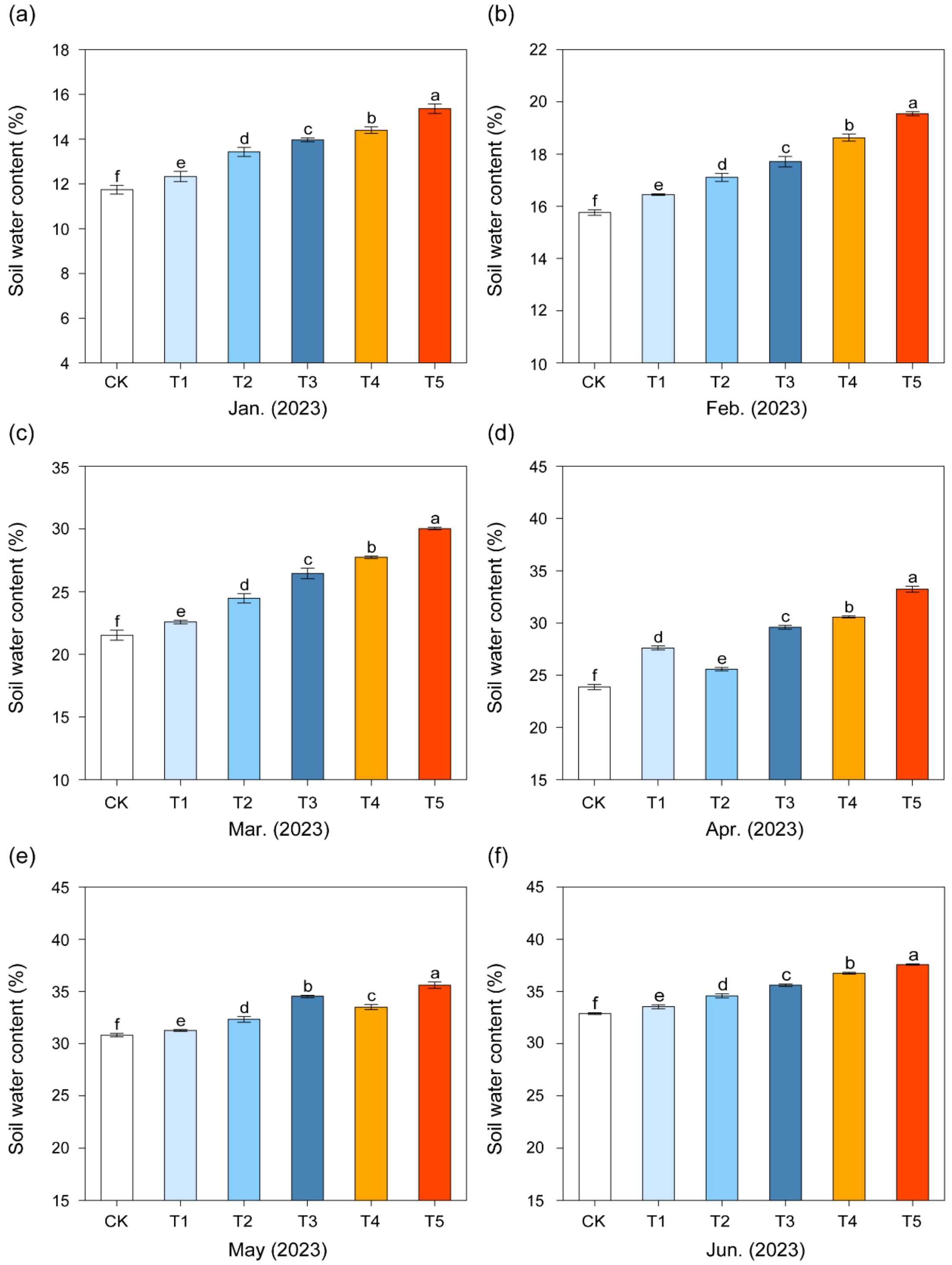

(a)
Soil water content (%)
CK
T1
T2
T3
T4
T5
Jan. (2023)
(b)
Feb. (2023)
(c)
Mar. (2023)
(d)
Apr. (2023)
(e)
May (2023)
(f)
Jun. (2023)

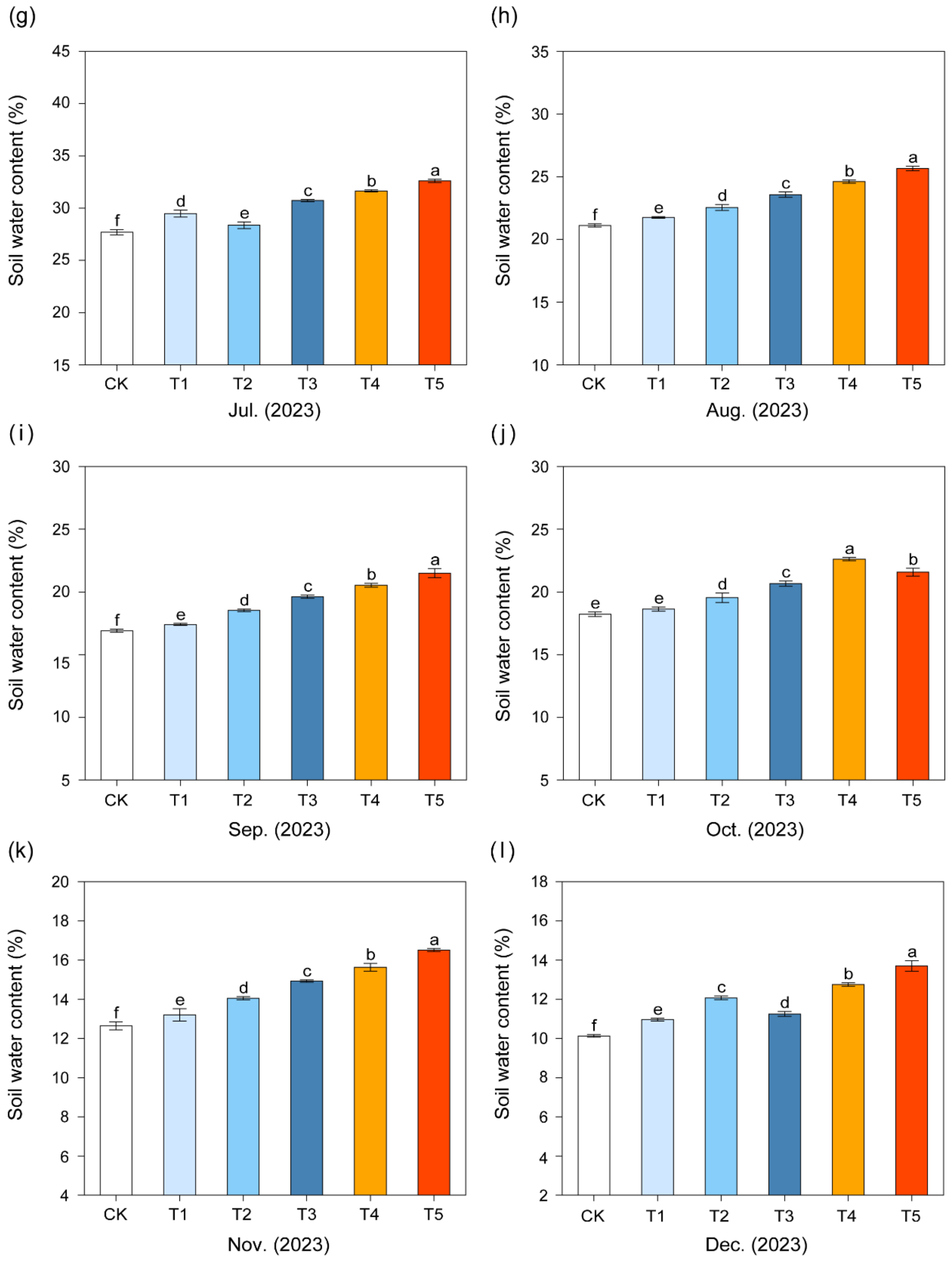
(g)
Soil water content (%)
CK T1 T2 T3 T4 T5
f d e c b a
Jul. (2023)
(h)
Soil water content (%)
CK T1 T2 T3 T4 T5
f e d c b a
Aug. (2023)
(i)
Soil water content (%)
CK T1 T2 T3 T4 T5
f e d c b a
Sep. (2023)
(j)
Soil water content (%)
CK T1 T2 T3 T4 T5
e e d c a b
Oct. (2023)
(k)
Soil water content (%)
CK T1 T2 T3 T4 T5
f e d c b a
Nov. (2023)
(l)
Soil water content (%)
CK T1 T2 T3 T4 T5
f e c d b a
Dec. (2023)

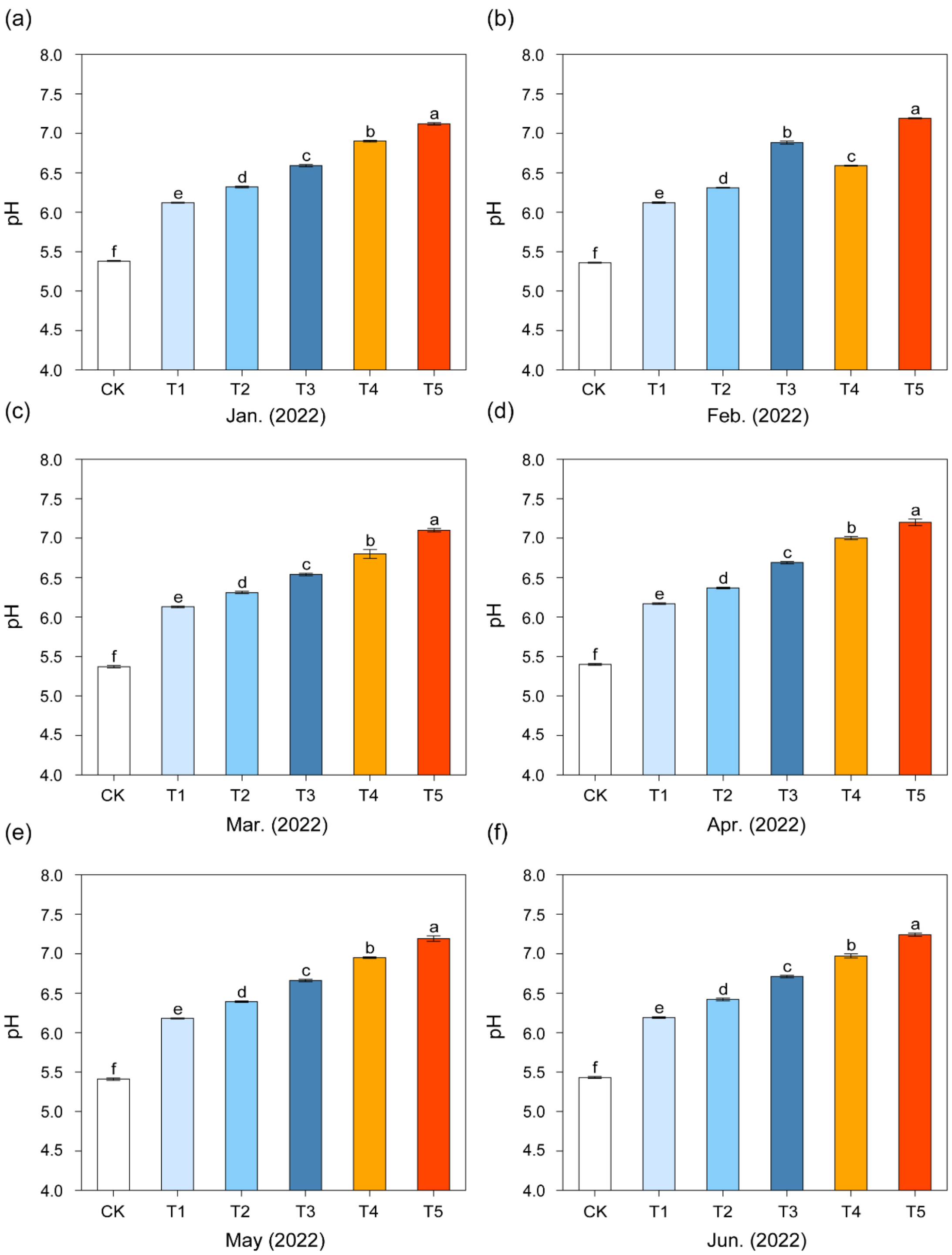

(a)
pH
8.0
7.5
7.0
6.5
6.0
5.5
5.0
4.5
4.0
f e d c b a
CK T1 T2 T3 T4 T5
Jan. (2022)
(b)
pH
f e d b c a
CK T1 T2 T3 T4 T5
Feb. (2022)
(c)
pH
f e d c b a
CK T1 T2 T3 T4 T5
Mar. (2022)
(d)
pH
f e d c b a
CK T1 T2 T3 T4 T5
Apr. (2022)
(e)
pH
f e d c b a
CK T1 T2 T3 T4 T5
May (2022)
(f)
pH
f e d c b a
CK T1 T2 T3 T4 T5
Jun. (2022)

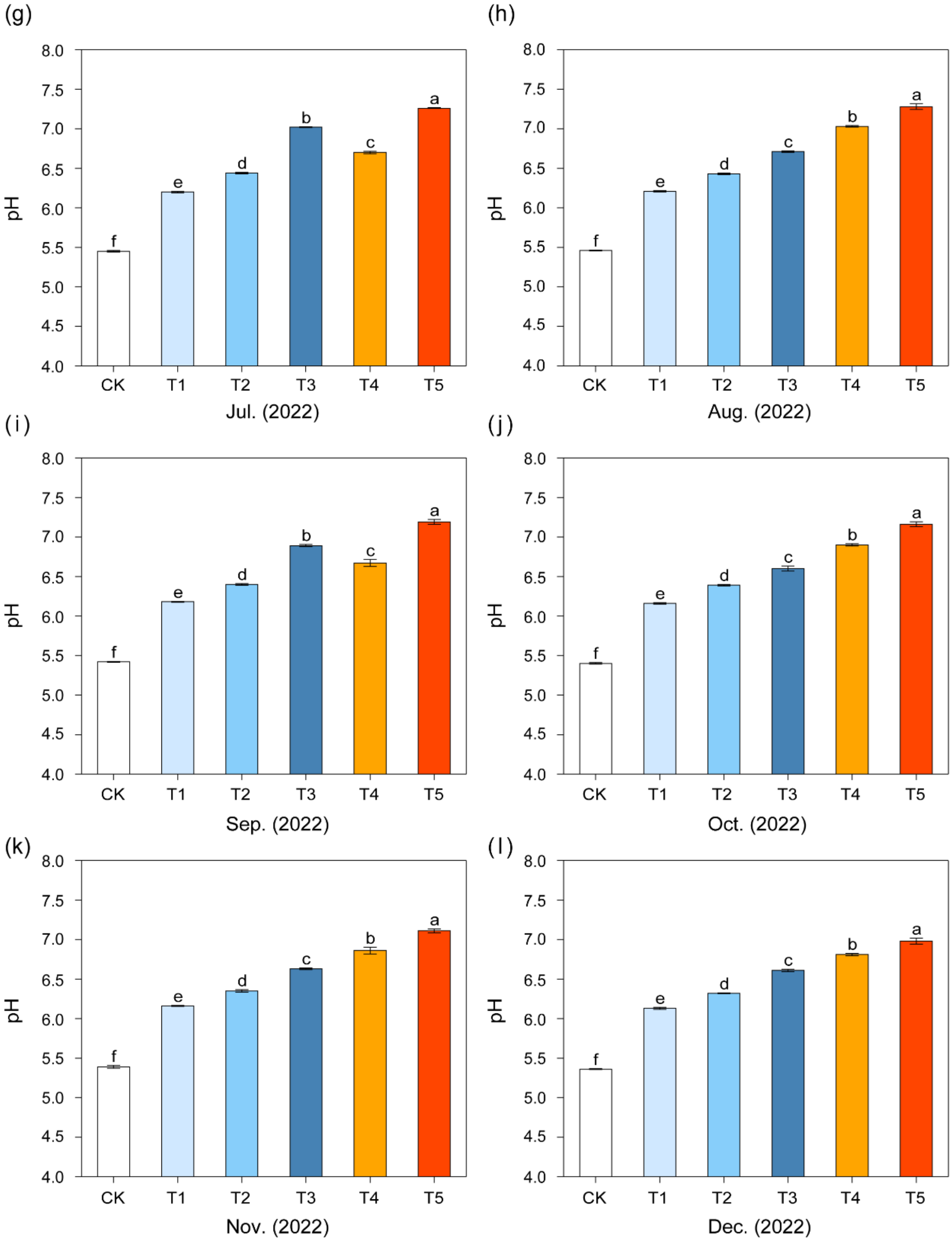
(g)
(h)
(i)
(j)
(k)
(l)
pH
8.0
7.5
7.0
6.5
6.0
5.5
5.0
4.5
4.0
CK
T1
T2
T3
T4
T5
a
b
c
d
e
f
Jul. (2022)
Aug. (2022)
Sep. (2022)
Oct. (2022)
Nov. (2022)
Dec. (2022)

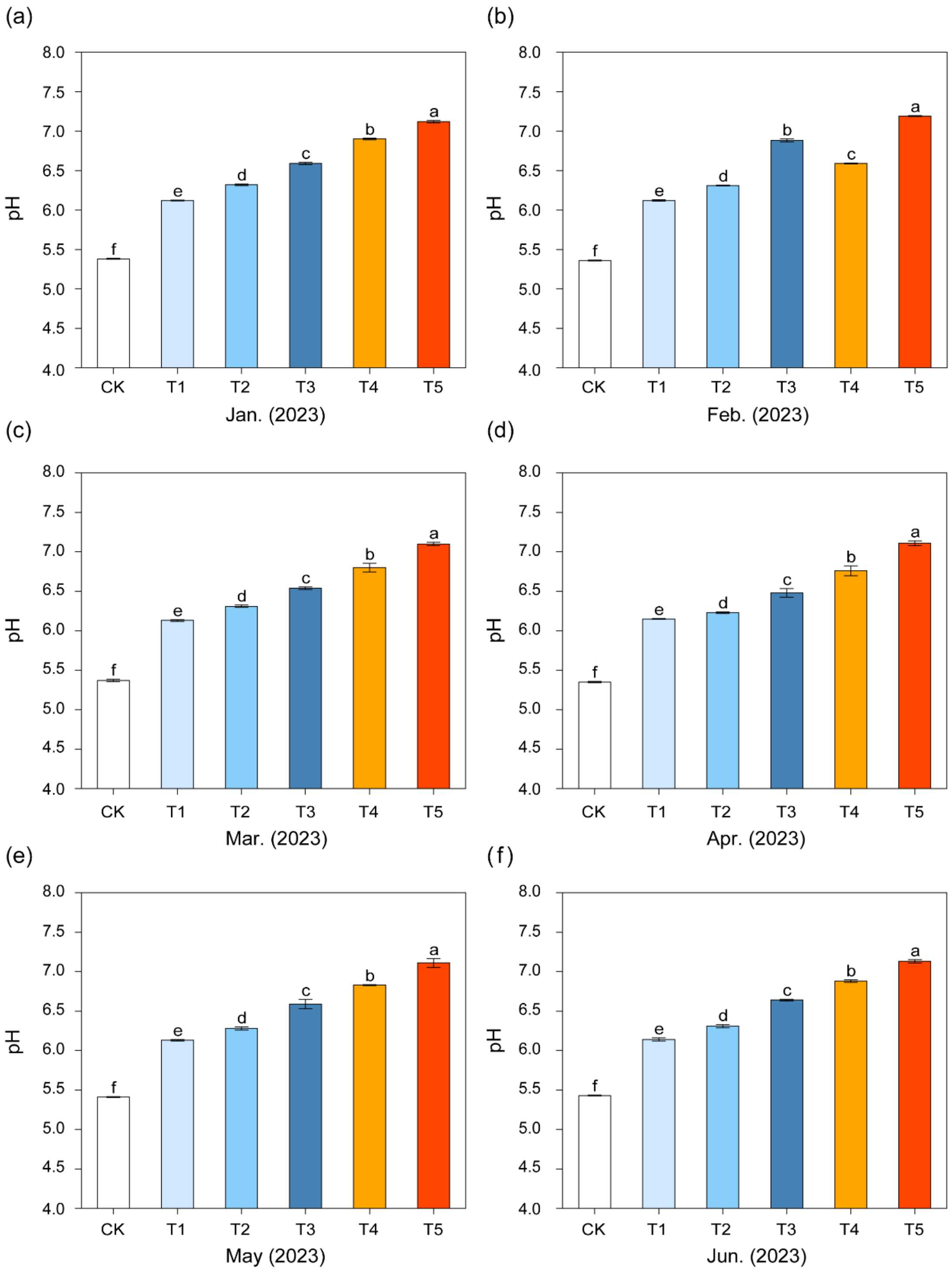
(a)
pH
8.0
7.5
7.0
6.5
6.0
5.5
5.0
4.5
4.0
f
e
d
c
b
a
CK
T1
T2
T3
T4
T5
Jan. (2023)
(b)
pH
f
e
d
b
c
a
CK
T1
T2
T3
T4
T5
Feb. (2023)
(c)
pH
f
e
d
c
b
a
CK
T1
T2
T3
T4
T5
Mar. (2023)
(d)
pH
f
e
d
c
b
a
CK
T1
T2
T3
T4
T5
Apr. (2023)
(e)
pH
f
e
d
c
b
a
CK
T1
T2
T3
T4
T5
May (2023)
(f)
pH
f
e
d
c
b
a
CK
T1
T2
T3
T4
T5
Jun. (2023)

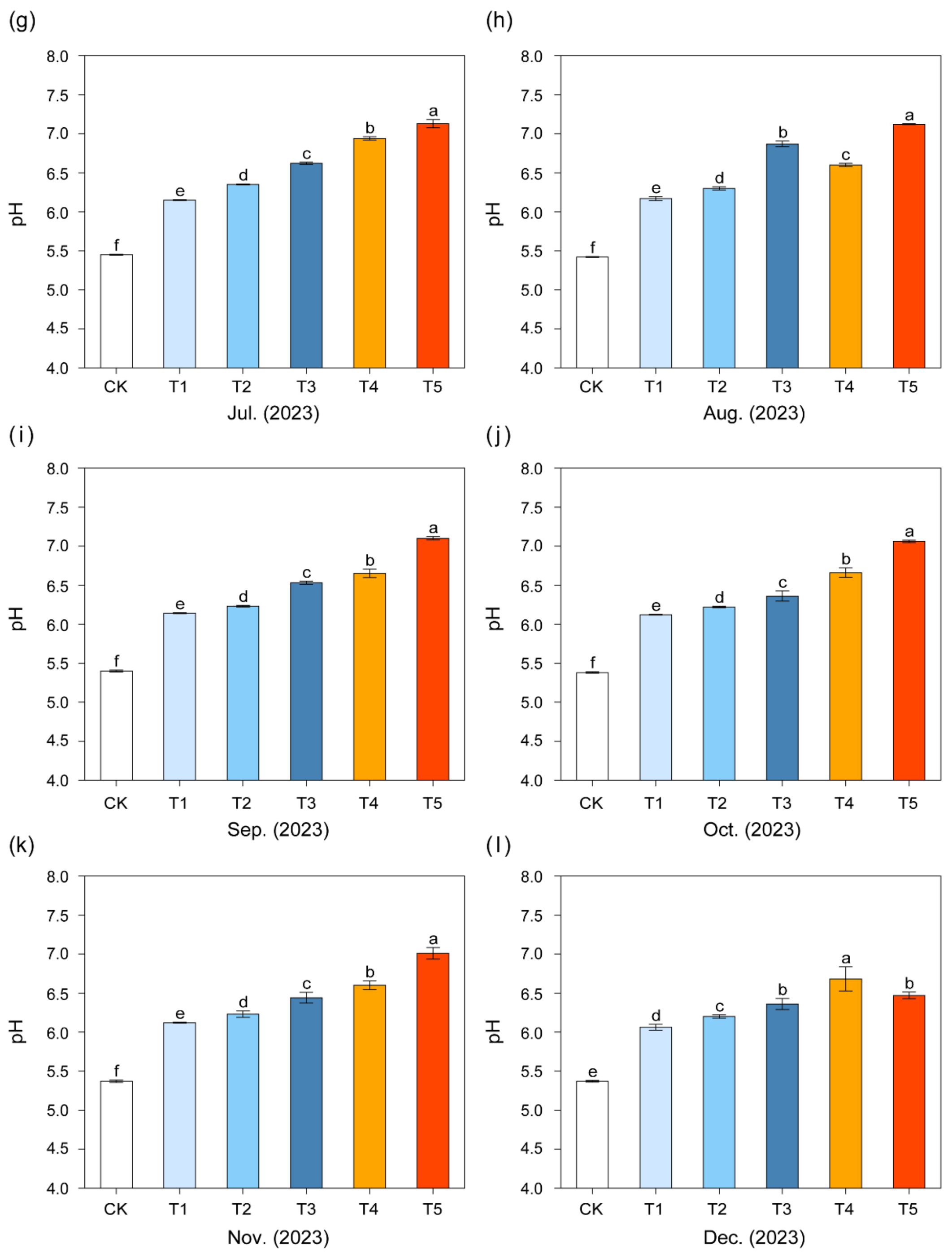

Figure S1. Monthly variations of soil temperature (ST), soil water content (SWC), and soil pH under different treatments in 2022 and 2023: (a) January; (b) February; (c) March; (d) April; (e) May; (f) June; (g) July; (h) August; (i) September; (j) October; (k) November; (l) December. Different lowercase letters indicate significant differences among treatments at $p < 0.05$.

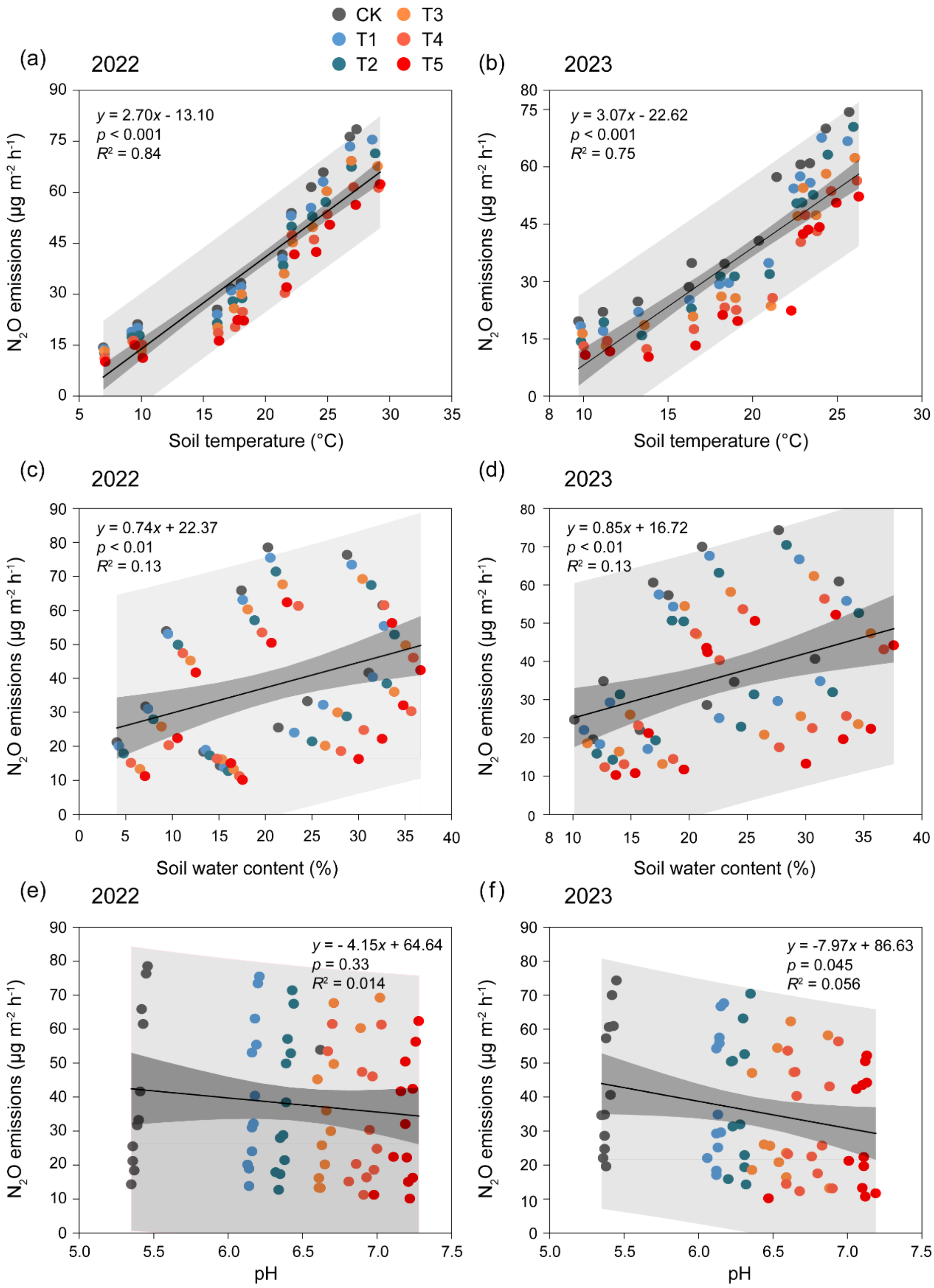

Figure S2. Relationship between N_2O emissions and ST, SWC, and pH in 2022 and 2023.
